# Muscle–Bone Relationship: Positive Association Between Appendicular Skeletal Muscle Mass and Bone Microarchitecture in Postmenopausal Women

**DOI:** 10.1155/jnme/3170619

**Published:** 2025-11-22

**Authors:** Camila Vilarinho Vidigal, Patrícia Paula da Fonseca Grili, Geise Ferreira da Cruz, Ben-Hur Albergaria, Luciane Daniele Cardoso, José Luiz Marques-Rocha, Valdete Regina Guandalini

**Affiliations:** ^1^Postgraduate Program in Nutrition and Health, Health Sciences Center, Federal University of Espírito Santo, Vitória, Espírito Santo, Brazil; ^2^Department of Nutrition, Health Sciences Center, Federal University of Espírito Santo, Vitória, Espírito Santo, Brazil; ^3^Department of Social Medicine, Federal University of Espírito Santo, Vitória, Espírito Santo, Brazil

**Keywords:** bone mass, menopause, muscle mass, osteoporosis, sarcopenia

## Abstract

**Background:**

Low estrogen levels affect bone mineral density and bone microarchitecture, increasing the risk of osteoporosis. This hormonal change can also contribute to the loss of strength, muscle mass, and physical performance.

**Aim:**

To investigate associations between the components of sarcopenia and bone microarchitecture in postmenopausal women.

**Methods:**

A cross-sectional study was conducted with 98 women ≥ 50 years of age. Data were collected on sociodemographic, lifestyle, and clinical characteristics. Measurements were performed of height, body mass, calf circumference adjusted for BMI, grip strength, and physical performance using the Timed Up and Go test. The appendicular skeletal muscle mass index (ASMI) and bone mineral density were estimated by dual energy X-ray absorptiometry. Bone microarchitecture was determined by the trabecular bone score. The association between ASMI and bone microarchitecture was tested using multivariate linear regression analysis with adjusted models.

**Results:**

The participants were predominantly between 60.0 and 69.9 years of age (62.3%), lived with a partner (51.0%), declared themselves to be non-White (59.2%), had low or no education (67.3%), were sufficiently active (52.0%), and did not consume alcohol (87.8%) or smoke (94.9%). ASMI was associated with bone microarchitecture after the adjustment of the models (*β* = 0.047; 95% CI: 0.009–0.084; *p* = 0.015).

**Conclusion:**

The positive association found between skeletal muscle mass and bone microarchitecture indicates that maintaining muscle mass may play a significant role in bone health among postmenopausal women.

## 1. Introduction

Menopause is part of the natural process of female aging and indicates the end of a woman's reproductive capacity [1]. This stage involves intense hormonal variations mainly due to the decline in estrogen, causing significant changes in body composition and bone mass [1, 2]. Such changes are related to metabolic disorders and a greater risk of osteoporosis [1, 2].

The most notable changes in body composition are the progressive reduction in strength and muscle mass, which can lead to the development of sarcopenia [2]. Sarcopenia is the simultaneous presence of low muscle strength, quantity, and quality and is considered severe when accompanied by low physical performance [3]. In a study conducted with postmenopausal Hungarian women over 50 years of age, the prevalence of sarcopenia was 31% [4], demonstrating the importance of diagnosing this condition.

In addition to changes in muscle tissue, there is also an increase in bone reabsorption, with the consequent development of osteoporosis, which is a disease characterized by low bone mineral density (BMD) and degraded bone microarchitecture [5, 6]. The assessment of bone microarchitecture is complementary to dual energy X-ray absorptiometry (DXA) in the identification of the risk of fractures [7, 8].

Bone microarchitecture can be indirectly determined through the trabecular bone score (TBS), which is an index calculated from the variation in pixel gray levels in DXA images of the lumbar spine and is associated with microarchitectural parameters obtained by quantitative computed tomography (QCT) and high-resolution peripheral QCT (HR-pQCT) [7, 9]. Pal et al. found that bone quality, but not quantity, was significantly lower among postmenopausal women with sarcopenic diabetes, even suggesting that assessing BMD alone may underestimate bone health in women with the simultaneous occurrence of these two conditions [10].

The effects of muscle mass on bone have been attributed to mechanical and endocrine interactions between these tissues, as muscle contraction is capable of causing changes in bone structure as well as stimulating the secretion of cytokines that favor osteogenesis [11–15]. Studies conducted with Chinese adults [16] and Belgian older people [17] revealed associations between bone microarchitecture and skeletal muscle mass and muscle strength, respectively. Both studies addressed the components of sarcopenia, but neither was conducted specifically with postmenopausal women.

Although the association between muscle and bone has been demonstrated, studies associating the components of sarcopenia with bone health are scarce, especially those analyzing bone microarchitecture in postmenopausal women. Therefore, the aim of the present study was to investigate associations between the components of sarcopenia and bone microarchitecture in postmenopausal women.

## 2. Methods

### 2.1. Study Design and Participants

An observational cross-sectional study was conducted at the menopause and osteoporosis outpatient clinic of a university hospital in southeastern Brazil between June 2019 and March 2020. Women over 50 years of age with clinically confirmed menopause—no menstrual period for 12 consecutive months—were included in the study.

Participants were selected through simple probabilistic sampling. Based on a previously described sample calculation method [18], the minimum sample required was 147 participants. Women enrolled at the outpatient clinic were contacted by telephone to participate in the study. In cases of refusal or noncompliance with the inclusion criteria, a new drawing was performed to replace the participant. Those for whom contact was not possible after three attempts, those undergoing hormone replacement therapy, those not submitted to the necessary assessments for the study, and those without a TBS were excluded.

**Figure 1:**
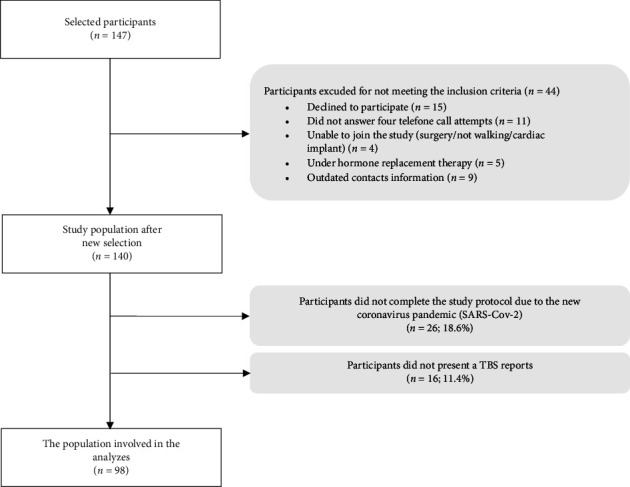
Flowchart of participant selection process.

A total of 147 women were selected to participate in this study, 44 of whom were excluded for not meeting the eligibility criteria. Although new participants were selected, the sampling process had to be interrupted due to the onset of the COVID-19 pandemic. A total of 140 women were selected, 26 of whom were unable to participate in the second stage of the study due to quarantine measures during the pandemic (18.6%), and 16 did not have a TBS report (11.4%). The final sample consisted of 98 participants (Figure 1).

### 2.2. Variables of Interest

Data were collected by qualified, duly trained interviewers. BMD and the appendicular skeletal muscle mass index (ASMI) were estimated using DXA. The exams were performed by a trained, certified radiology technician using the GE Lunar Prodigy Advance device, and the data were interpreted and reported by a single physician.

### 2.3. Outcome Variable

Bone microarchitecture was the outcome variable and was assessed indirectly using the TBS iNsight software (version 3.0.3.0; Medmaps) for the analysis of DXA images of the lumbar spine (between the L1 and L4 vertebrae) to obtain the TBS. The TBS was derived from the average of the four vertebrae analyzed and was considered a continuous variable in the analysis.

### 2.4. Exposure Variables

The anthropometric variables that compose the sarcopenia phenotype were considered exposure variables. Body mass (kg) and height (cm) were measured using recommended methods [19]. Body mass index (BMI) was calculated considering the ratio between current body mass (kg) and height squared (m^2^). Calf circumference (CC) was measured (cm) at the widest part of the calf muscle [20] and adjusted for BMI [21].

Muscle quantity was assessed based on the ASMI (kg/m^2^), which was determined by DXA. For this exam, the participants fasted for 4 h and wore light clothing in compliance with recommendations for measurement accuracy [22].

Muscle strength was determined based on grip strength, and physical performance was assessed using the Timed Up and Go (TUG) test. Grip strength was measured three times on the dominant hand using a Jamar handgrip dynamometer, following the method recommended by the American Association of Hand Therapy. The highest value of the three measurements was considered for analysis [23]. The TUG test (stand up from a chair, walk 3 m, turn around, walk back to the chair, and sit down again) was also performed in triplicate, with the average time of the three trials used for analysis [24].

### 2.5. Covariates

Sociodemographic variables (age, marital status, skin color, and schooling), lifestyle characteristics (alcohol consumption, smoking status, and physical activity level), and clinical history (time since menopause, disease diagnosis, and use of calcium supplements, vitamin D, and medications that affect bone metabolism) were collected using a questionnaire. The participants were classified into three age groups (50–59.9 years, 60–69.9 years, and ≥ 70.0 years) and based on marital status (with or without a partner). Skin color was self-declared according to the classification of the Brazilian Institute of Geography and Statistics and subsequently categorized into white or non-White [25]. Schooling was classified as “no or low” (when illiterate, with incomplete or complete primary school, or incomplete high school), high school (when complete and incomplete higher education), and higher education (when complete) [26].

Physical activity level was assessed using the long version of the International Physical Activity Questionnaire (IPAQ), which has been validated for the Brazilian population [27]. Women who practiced at least 150 min of physical activity per week were considered “sufficiently active” [28]. Alcohol consumption and smoking were categorized as “yes” or “no”, considering the participant's habit at the time of the study.

Time since menopause was calculated based on the difference between the participant's age at the time of the interview and the age at which menopause occurred. The median time since menopause was considered for analysis. The diagnosis of diseases (diabetes mellitus, high blood pressure, arthritis/arthrosis, etc.), use of medications that alter bone metabolism, and the consumption of calcium and vitamin D supplements were self-reported during the interview. BMD was obtained by DXA, preferably of the lumbar spine (between L1 and L4). If assessing the lumbar spine was not possible, the right femoral neck was assessed. Women were diagnosed with osteoporosis when the T-score was ≤ −2.5 SD and with osteopenia when T-scores fell between −1 SD and −2.5 SD. Those with a T-score ≥ −1 SD were considered to have normal BMD [5, 6].

### 2.6. Ethical Aspects

Participation was voluntary and consent was provided in writing by signing the statement of informed consent, in accordance with Resolution 466/12 of the National Board of Health, Ministry of Health [29]. This study received approval from the Human Research Ethics Committee of the Federal University of Espírito Santo (protocol number: 88131818.0.0000.5060; approval certificate number: 2.621.794).

### 2.7. Data Analysis

Statistical analysis was conducted using the Statistical Package for the Social Sciences (SPSS) for Windows, version 25.0. One-way ANOVA and the Student's *t*-test were used to compare the distribution of categorical sociodemographic variables and clinical conditions within the sample according to mean bone microarchitecture. The Kolmogorov–Smirnov test was used to determine the normality of the variables. Pearson's correlation analysis was applied to test the strength of correlations between two continuous variables. Multivariate linear regression was conducted with four adjusted models to investigate associations between exposure variables and TBS and estimate odds ratios for the outcomes according to the study variables. Variables with a *p* value < 0.05 on the one-way ANOVA test, Student's *t*-test, and Pearson's correlation analysis as well as those related to bone health were included in the regression. The analysis was adjusted for age (years), skin color, marital status, and schooling (Model 1); Model 2: Model 1 + time since menopause (years), alcohol intake, smoking status, and physical activity level; Model 3: Model 2 + calcium supplementation, vitamin D supplementation, and medications that affect bone metabolism; Model 4: Model 3 + diabetes mellitus, arterial hypertension, arthritis/arthrosis, and BMI. No collinearity was found between variables (VIF > 10) and the Durbin–Watson value was 2. A significance level of 5% was adopted for all analyses.

## 3. Results

Ninety-eight women participated in the study. Those between 60.0 and 69.9 years of age (62.3%), who lived with their partners (51.0%), were non-White (59.2%), had little or no schooling (67.3%), were sufficiently active (52.0%), and had never consumed alcohol (87.8%) or smoked (94.9%) predominated in the sample (Table 1). The mean TBS differed significantly across age categories (*p*=0.023). Women ≥ 70.0 years of age had significantly lower mean TBS compared to those up to 69.9 years of age (Table 1).

In the analysis of health conditions, time since menopause, use of calcium and vitamin D supplements, and medications that alter bone metabolism, associations were found between the mean TBS and arthritis/arthrosis (*p*=0.041), BMD (*p*=0.032), and vitamin D supplementation (Table 2).

Considering mean values of BMI, CC adjusted for BMI, maximum grip strength, ASMI, and results of the TUG test, the sample had excess weight, adequate CC, preserved muscle strength and mass, and physical performance within normal limits. Mean TBS values were indicative of partially degraded bone microarchitecture (Table 3).

**Figure 2:**
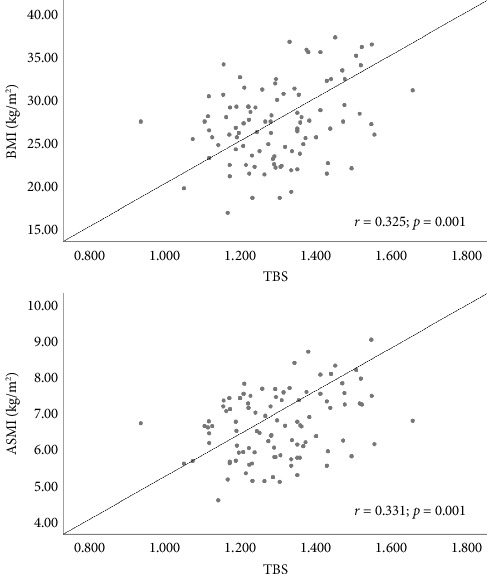
Correlation between body mass index and appendicular skeletal muscle mass index with trabecular bone score. Pearson correlation test; BMI: body mass index; ASMI: appendicular skeletal muscle mass index.

No statistically significant correlations were found between the TBS and maximum grip strength (kg) (*r* = −0.031; *p*=0.764), the TUG test result (s) (*r* = 0.068; *p*=0.504), and CC adjusted for BMI (cm) (*r* = 0.160; *p*=0.117). Weak positive correlations were observed between the TBS and BMI (kg/m^2^) (*r* = 0.325; *p*=0.001) and ASMI (kg/m^2^) (*r* = 0.331; *p*=0.001) (Figure 2).

In the multivariate linear regression analysis considering bone microarchitecture as the dependent variable and using adjusted models, only ASMI remained associated with bone microarchitecture. The model explained 33% of the variance in the outcome (*R*^2^ = 0.33). Among the predictor variables, a significant association with the ASMI was found (*β* = 0.047; 95% CI: 0.009–0.084; *p*=0.015). Each 1 kg/m^2^ increase in ASMI was associated with a 0.047-point increase in the TBS (95% CI: 0.009–0.084; *p*=0.015) (Table 4).

## 4. Discussion

The results of the present study revealed a positive association between ASMI and TBS values. Previous studies have reported the relationship between muscle mass and bone health indicators [16, 30]. Qi et al. investigated the relationship between the components of sarcopenia and osteoporosis in 203 Chinese women (mean age: 67.4 years) and found positive associations between relative appendicular skeletal muscle mass and bone microarchitecture after the adjustment of the models by age, BMI, smoking, alcohol intake, hypertension, and Type 2 diabetes mellitus [16]. The study also revealed that sarcopenic women had significantly lower TBS and BMD values than nonsarcopenic women. In a year-long analysis of 232 older people of both sexes (average age: 75.5 years), Locquete et al. found associations between the ASMI and the decline in TBS values, in addition to identifying a fivefold greater risk of developing osteoporosis in older people with sarcopenia [30].

This association may be attributed to mechanical and molecular foundations [11–15]. One of the possible mechanisms underlying the mechanical effect of muscle tissue in preserving BMD and bone microarchitecture is related to the presence of mechanical signal receptors in osteocytes [15]. When activated, these receptors contribute to the transduction of these signals to bone lining cells and osteoblasts, which favors bone renewal [15].

Mechanotransduction begins with the passage of mechanical signals from the extracellular to the intracellular environment [15]. Integrins receive stimuli and adjust their conformation to the extended position, connecting the cytoskeleton to the extracellular matrix through ligands involved in the process of cell differentiation and proliferation [15, 31]. Calcium channels open, enabling calcium to flow through, which converts mechanical signals into chemical signals and thereby activates a series of chemical reactions [15]. In turn, the opening of gap junctions enables the passage of signaling molecules of mechanical stimuli to the surrounding cells [15].

If muscle and bone communicate physiologically, this integration is reflected in the overlap of musculoskeletal diseases, such as sarcopenia and osteoporosis, which often coexist in a condition denominated osteosarcopenia [14]. A recent meta-analysis indicated that the prevalence of these conditions among Brazilians is 8.7% and that osteosarcopenia is associated with relevant clinical outcomes, such as a greater risk of falls, fractures, and mortality [32].

No associations were found between bone microarchitecture and grip strength or physical performance assessed using the TUG test in the present study, which is in agreement with data described by Seaton et al. in a predominantly female older population. One possible explanation is that the TBS assesses a central skeletal structure, whereas grip strength and the TUG test involve peripheral structures [33]. Another hypothesis is that the sample consisted of healthy, sufficiently active women who were regularly monitored by the healthcare system.

This study sheds light on the relationship between muscle tissue and bone health from the perspective of bone quality, which remains underexplored in the literature. The strengths of the study include the careful sample selection process and the use of DXA, which is considered the gold standard for assessing BMD and a reference for the determination of body composition. Although we did not exclude women who took medications that alter bone metabolism, adjustments were made to minimize the interference of this variable in our analyses. Moreover, the literature suggests a loss of sensitivity in the assessment of bone microarchitecture using TBSs in individuals with a BMI lower than 15 kg/m^2^ and higher than 37 kg/m^2^ [34], which was considered in the present study. As the sample did not include participants with BMI values in these ranges, this was not designated as an exclusion criterion.

This study also has limitations that should be considered. The participants were served by the Brazilian public healthcare system and often receive multidisciplinary treatment. Thus, the sample does not portray the Brazilian population in its entirety. However, this information clarifies some of the results, such as the majority of participants being self-declared Black or Brown, sufficiently active, did not consume alcohol, and did not smoke. Furthermore, the prevalence of diabetes and arthritis/arthrosis was low, with most participants taking calcium and vitamin D supplements. Another limitation regards the impossibility of inferring cause-and-effect relationships due to the cross-sectional design. Despite this, we emphasize the importance of preserving muscle mass during female aging, given the association between muscle and bone tissue observed in this study. Furthermore, the scarcity of studies analyzing the relationship between the components of sarcopenia and bone microarchitecture, especially the TBS, may have limited the discussion of the results. Few studies have employed this approach in the context of the Brazilian population.

## 5. Conclusion

The association between skeletal muscle mass and bone microarchitecture underscores the importance of integrated approaches aimed not only at bone health, but also at preserving and strengthening skeletal muscle mass as a fundamental part of preventive care for postmenopausal women.

## Figures and Tables

**Table 1:** Distribution of trabecular bone score means according to characterization of the sample categories of the evaluated women.

Variable (*n* = 98)	*n* (%)	TBS	*p* value
		Mean ± SD	
Age (years)	98 (100.0)		
Age group (years)^1^			**0.023**
50.0–59.9	11 (11.2)	1.388 ± 0.160	
60.0–69.9	61 (62.3)	1.309 ± 0.128	
≥ 70.0	26 (26.5)	1.263 ± 0.099	
Marital status^2^			0.155
With partner	50 (51.0)	1.324 ± 0.135	
Without a partner	48 (49.0)	1.286 ± 0.120	
Skin color^2^			0.821
White	40 (40.8)	1.309 ± 0.135	
Non-White	58 (59.2)	1.303 ± 0.126	
Schooling^1^			0.124
No or low	66 (67.3)	1.304 ± 0.124	
High school	22 (22.5)	1.278 ± 0.149	
College graduate	10 (10.2)	1.378 ± 0.095	
Alcohol consumption^2^			0.170
No	86 (87.8)	1.311 ± 0.128	
Yes	12 (12.2)	1.257 ± 0.133	
Smoking status^2^			0.611
No	93 (94.9)	1.307 ± 0.131	
Yes	5 (5.1)	1.277 ± 0.078	
Physical activity level^2^			0.289
Sufficiently active	51 (52.0)	1.292 ± 0.129	
Insufficiently active	47 (48.0)	1.320 ± 0.128	

*Note:* The bold value indicates *p* < 0.05.

^1^One-way ANOVA test.

^2^Student's *t*-test.

**Table 2:** Distribution of trabecular bone score means according to clinical conditions categories of the evaluated women.

Variable (*n* = 98)	*n* (%)	TBS	*p* value
		Mean ± SD	
Diabetes mellitus^1^			**0.009**
No	75 (76.5)	1.323 ± 0.122	
Yes	23 (23.5)	1.243 ± 0.135	
Arterial hypertension^1^			0.762
No	50 (51.0)	1.309 ± 0.136	
Yes	48 (49.0)	1.301 ± 0.122	
Arthritis/arthrosis^1^			**0.041**
No	75 (76.5)	1.353 ± 0.134	
Yes	23 (23.5)	1.290 ± 0.125	
BMD^2^			**< 0.001**
Normal	14 (14.3)	1.444 ± 0.113^a^	
Osteopenia	47 (48.0)	1.298 ± 0.122^b^	
Osteoporosis	37 (37.7)	1.262 ± 0.108^b^	
Calcium supplementation^1^			0.117
No	31 (31.6)	1.335 ± 0.146	
Yes	67 (68.4)	1.291 ± 0.119	
Vitamin D supplementation^1^			**0.032**
No	37 (37.7)	1.341 ± 0.137	
Yes	61 (62.3)	1.284 ± 0.120	
Medications that affect bone metabolism^1^			0.172
No	49 (50.0)	1.323 ± 0.146	
Yes	49 (50.0)	1.287 ± 0.108	
Time since menopause (years)^1^			0.116
≤ 19	50 (51.0)	1.325 ± 0.141	
> 19	48 (49.0)	1.284 ± 0.111	

*Note:* Different letters indicate differences between tests. The bold values indicate *p* < 0.05.

^1^Student's *t*-test.

^2^One-way ANOVA test.

**Table 3:** Average of anthropometric variables and sarcopenia components among the women evaluated.

Variable (*n* = 98)	Mean ± SD
BMI (kg/m^2^)	27.13 ± 4.52
CC adjusted for BMI (cm)	33.63 ± 2.81
Maximum grip strength (kg)	24.09 ± 5.44
ASMI (kg/m^2^)	6.66 ± 0.92
TUG test (s)	11.74 ± 3.66
TBS	1.305 ± 0.128

*Note:* Continuous variables were presented as means and standard deviations (SD). ASMI: appendicular skeletal muscle mass index.

Abbreviations: BMI, body mass index; CC, calf circumference; TBS, trabecular bone score; TUG test, Timed Up and Go test.

**Table 4:** Association between trabecular bone score and appendicular skeletal muscle mass index based on multivariate linear regression.

Variable	Crude model			Model 1			Model 2			Model 3			Model 4		
	*β*	CI (95%)	*p* value	*β*	CI (95%)	*p* value	*β*	CI (95%)	*p* value	*β*	CI (95%)	*p* value	*β*	CI (95%)	*p* value
Maximum grip strength	−0.002	−0.007–0.003	0.444	−0.002	−0.007–0.003	0.363	−0.002	−0.007–0.003	0.373	−0.003	−0.008–0.002	0.210	−0.003	−0.007–0.002	0.228
ASMI	0.048	0.020–0.076	**0.001**	0.041	0.012–0.069	0.006	**0.040**	0.011–0.069	**0.007**	0.040	0.003–0.077	**0.032**	0.047	0.009–0.084	0.015
TUG	0.000	−0.007–0.008	0.901	0.002	−0.006–0.010	0.628	0.002	−0.006–0.010	0.662	0.003	−0.005–0.010	0.456	0.003	−0.005–0.011	0.442

*Note:R* ^2^ = 0.33. Legend: ASMI: appendicular skeletal muscle mass index. TUG: Timed Up and Go test. Model 1: Adjusted for age (years), skin color, marital status and schooling; Model 2: Model 1 + alcohol consumption, smoking status and physical activity level; Model 3: Model 2 + calcium supplementation, vitamin D supplementation and medications that affect bone metabolism; Model 4: Model 3 + diabetes mellitus, arterial hypertension, arthritis/arthrosis and BMI.

## Data Availability

The datasets generated during and/or analyzed during the current study are available from the corresponding author on reasonable request.
